# Factors related to knowledge and perception of women about smoking: a cross sectional study from a developing country

**DOI:** 10.1186/1472-6874-11-16

**Published:** 2011-05-24

**Authors:** Seema Bhanji, Marie Andrades, Fawad Taj, Ali K Khuwaja

**Affiliations:** 1 Department of Family Medicine, The Aga Khan University, Stadium Road, PO Box 3500, Karachi - 74800, Pakistan; 2 The Aga Khan University, Stadium Road, PO Box 3500, Karachi - 74800, Pakistan; 3 Department of Community Health Sciences, The Aga Khan University, Stadium Road, PO Box 3500, Karachi - 74800, Pakistan

## Abstract

**Background:**

Smoking rates among women are currently low, but they are the fastest growing segment of cigarette smoking population in developing countries. We aimed to assess the knowledge and perceptions towards smoking and to identify the factors related with level of knowledge and perceptions among adult women in urban slums.

**Methods:**

This was a cross sectional study conducted on 250 adult (≥18 years of age) women attending primary care clinics in three slums of Karachi, Pakistan. A pre-tested and structured, interviewer administered questionnaire was used for data collection. Factors associated with level of understanding about smoking were analyzed with chi-square test.

**Results:**

Most of the women knew that smoking has adverse effects on women and children's health but the knowledge of specific health effects was limited. About one third of the women knew that active smoking can cause lung disease, but only a small percentage (7%) knew that it could lead to heart disease. None of the women were aware that smoking contributes to infertility and osteoporosis. A small proportion of women were aware that smoking can lead to low birth weight (7%), congenital anomalies (5%) and less than 1% of women knew that it contributes to pregnancy loss, still birth and preterm delivery. The understanding of passive smoking affecting children's lung was low (20%) and a similar proportion voiced concern about the bad influence of maternal smoking on children. Educated women had better knowledge of health effects of smoking. Education was associated with having better knowledge about effects on women health in general (p = 0.02) and specific effects like lung (p = 0.03) and reproductive health effects (p < 0.001). Education was also associated with knowledge regarding effects on fetus (p < 0.001) and children (p < 0.005). Although most of the women disliked being around smokers, more than one third thought that smoking decreases boredom (39%), tension (38%) and also helps to relax (40%). A large proportion (48%) of women had the misconception that smoking helps to reduce weight.

**Conclusions:**

This study reveals that women are aware of the general ill effects of smoking but fail to identify smoking to be associated with female maladies particularly those who were illiterate and had lower levels of education. Understanding and attitudes needs to be improved by increasing health awareness and education of women in these urban communities with special emphasis on the effects of smoking on women's health.

## Background

While the prevalence of smoking has been slowly declining in the developed countries over the past 20 years, smoking prevalence rates have been steadily increasing in developing countries. It is projected that total tobacco-attributable deaths will rise from 5.4 million in 2005 to 6.4 million in 2015 and 8.3 million in 2030 with 80% of these additional deaths occurring in developing nations [1]. Tobacco-attributable deaths are projected to further decline by 9% between 2002 and 2030 in high-income countries, but to double from 3.4 million to 6.8 million in low- and middle income countries [2].

Smoking prevalence rates among men in developing countries are high; current prevalence among women is low but is projected to rise. According to World Health survey, the prevalence of current tobacco smoking in Pakistan is 19.1% (32.4 in males vs. 5.7% in females) [3]. However, there is a trend among young women towards taking up smoking despite cultural and religious obstacles [4]. A rise in tobacco use by young schoolgirls is a danger signal because those who start as children find it hardest to quit. Also there is a dose dependent relationship of many of these diseases with smoking.

Women have health risks and consequences of smoking that are specific to their gender. The problems unique to their gender pertain to their reproductive and non-reproductive function. The health risks of smoking among women are mainly osteoporosis, infertility, cervical cancer and pregnancy related problems (miscarriage, low birth weight, ectopic pregnancy, perinatal mortality and Sudden Infant Death Syndrome (SIDS) [5-8]. Besides their unique problems, diseases common to both genders are also on the rise in women. Lung cancer, once rare among women, has surpassed breast cancer as the leading cause of female cancer death in the United States, now accounting for 25% of all cancer deaths among women. Smoking is a major cause of coronary heart disease among women [9]. For women younger than 50 years, the majority of coronary heart disease is attributable to smoking. Cigarette smoking is a primary cause of chronic obstructive pulmonary disease (COPD) among women, and the risk increases with the amount and duration of smoking. Approximately 90% of mortality from COPD among women in the United States can be attributed to cigarette smoking [9].

Besides active smoking, exposure to passive smoking or second hand smoke (SHS) is a big threat to the health of women, fetus and children [10]. The estimated prevalence of passive smoking in women globally is found to be 37 to 70% [11], depending on the definition of passive smoking used, with the highest exposure to women of reproductive age group. Effects of passive smoking on children's health are also a growing concern. A US study reported 60% of children to be exposed to passive smoking [12], the primary source of passive smoking being home.

Theoretical models have suggested that besides many other factors, knowledge plays an important role in initiating the process of behavior change. For any intervention to be effective, knowledge of adverse effects is essential as it facilitates the progression of a person from pre-contemplation to contemplation phase of the behavioral cycle, where the decision for action (reduce exposure to SHS or quitting smoking) can be taken [13]. Research has demonstrated that knowledge about health risks of smoking is a preventive factor for taking up tobacco smoking [11]. In addition better knowledge leads to lesser tolerance and reduce exposure to second hand smoke [14-16] The models for intervention for ETS have also suggested a two level approach; Individual and community level. Nisar et al found that illiterate women in a community sample in Pakistan were more likely to consume tobacco as compared to women with more than five years of schooling [17]. Data shows that women belonging to low socioeconomic strata tend to have less education and hence are less informed about the harmful effects of smoking on self and others [18]. International Tobacco Control surveyed four high income countries (US, UK, Canada and Australia) and showed that higher income and education are associated with better knowledge [19]. Therefore it is important to investigate the level of knowledge of women belonging to low socioeconomic strata.

Literature regarding knowledge of health effects of active and passive smoking is limited for our population [20,21]. We planned to investigate the knowledge and perceptions towards smoking among adult women in three urban slum settlements in Karachi, Pakistan. We also studied factors related to knowledge and perceptions towards smoking.

## Methods

This was a cross sectional study conducted at three slums of Karachi, the largest city and economic capital of Pakistan. These slums are almost identical to the other slums of Karachi, where a substantially large population is residing. Being a questionnaire-based study, no ethical issue was expected, nevertheless this study was reviewed by our departmental research committee and permission was also obtained from the administration of studied clinics. Face to face interviews were conducted for data collection by medical students trained for the purpose. The interviewers approached individuals presenting to the primary care clinics at three urban slums of Karachi. After explaining the objectives, ensuring confidentiality of response and the right to not participate without any adverse consequences, all the participants were asked to give consent. All women of 18 years and above attending primary care clinics as a patient or attendants were eligible for inclusion to the study. In all, 310 individuals were approached consecutively as being eligible for inclusion in the study. The response rate was 80.6% resulting in a total of 250 participating women.

Extensive literature search of article containing key words "women", "tobacco", "health effects of environmental tobacco smoke" was performed. Key papers on the subject e.g. papers by Roth LK [22], Griffiths AN [23], Helgason [16] and Ma Gx [14,15] were found to be particularly helpful. Questionnaires used in these studies were reviewed by fellow investigators and a modified questionnaire was developed from consensus by investigators. The designed questionnaire was also reviewed by an expert for content and face validity. It comprised of three sections: demographic variables, knowledge variables and perception variables. The demographic variables were age, martial and education status, parity, average household income. The number of smokers in the household, relationship and time spent with smokers was also inquired. Knowledge regarding health effects of smoking of women on their own health, effect of smoking on pregnancy, on the fetus and effects of passive smoking on children were enquired. Questions on perceptions regarding smoking were also assessed. Questions included to assess the knowledge were: Is smoking harmful?, Does smoking cause cancer?, Does smoking cause heart diseases?, Does smoking cause lung disease?, Does smoking have ill effects on fetus?, Does passive smoking have ill effects on children?, Does passive smoking cause lung disease in children?, Does smoking have a bad influence on children?, Does occasional smoking have ill effects? Is smoking associated with infertility? and is smoking associated with osteoporosis?. While the questions included for assessing the perceptions were: Does smoking relieve boredom?, Does smoking cause relaxation?, Does smoking relieve stress? and Does smoking help in losing weight?

The questionnaire was kept anonymous. The questionnaire was developed in English. It was first translated in Urdu (National language of Pakistan) and then retranslated in English. Urdu version of the questionnaire was used for data collection. Questionnaire was pretested on a group of women before the data collection. Reponses were noted as Yes, No and Don't Know. The average time of interview was between 10-15 minutes.

The data was entered utilizing Epi Data 3.2 and was validated through double entry. Statistical Package for Social Sciences (SPSS) version 17.0 was used for statistical analysis. For analysis, No and don't know were combined as the proportion responding as don't know were small. Descriptive statistics (percentages) were calculated to determine the characteristics of the sample. Association between knowledge and perceptions towards smoking with age, income, educational status and presence smoker in the family was analyzed with chi-square test. A p-value of < 0.05 was taken as significant.

## Results

General characteristics of the study participants are described in table 1. Majority of the study participants were older than 25 years. Over half of these women (53%) had not received any formal education and a large majority of them (74%) had monthly household income of up to 5000 Pakistani Rupees (~84 USD). Almost a third of the study participants admitted having one or more smoker in the house which was most commonly the husband.

**Table 1:** General characteristics of the studied women

Characteristic	Number	Percentages
**Age groups**		
- 18 - 25 years	109	43.6
- 26 - 35 years	74	29.6
- 36 years and above	67	26.8

**Educational status**		
- No formal education	124	53.0
- Up to 5 years of schooling	34	14.6
- 6 - 10 years of schooling	66	28.3
- More than 10 years of schooling	26	11.1

**Monthly Income (Pak Rupees)**		
- Up to 5000.00	187	74.4
- More than 5000.00	63	25.6

**Smoker in the house**		
- Yes	75	30.0
- No	175	70.0

Only 2.0% women admitted smoking cigarettes. They smoked an average of 2.75 cigarettes per day and had been smoking for a mean duration of 13.5 years. In all, there were 261 children living in households having at least one smoker (Data has not been shown).

Most of the women (84%) reported smoking to have a bad effect on women's health. When asked about the specific adverse health effects, only one third of them knew it can cause some kind of respiratory problem, 14% of them reported that it can cause some kind of cancer and a very small percentage (7%) responded that it may lead to some heart problem. None of the women knew that it can lead to infertility or osteoporosis. Almost half of them did not think or were not sure if smoking occasionally has any effects. When effects of smoking on fetus was inquired, out of the total 77% of women knew it could have a bad effect on the fetus, while18% were not sure if smoking has any effect on the fetus and 5% reported that it does not affect the fetus at all. When asked more specifically about type of effects, the responses were small baby (7%), abnormal baby (5%) and respiratory problems (14%). A very small percentage knew it could lead to pregnancy loss (0.4%), still birth (0.4%) or preterm delivery (0.4%). When asked about knowledge of effects of passive smoking on children, a large proportion (88%) responded that it affects children's health negatively. On inquiry about specific diseases, about one fifth of them knew that it affects lungs in the form of asthma, persistent cough or breathing problems and similar proportion (19%) had concerns that it can have a bad influence on children.

Factors associated with knowledge about health effects of smoking are mentioned in table 2. Association of knowledge of health effects of smoking on women with age, educational status, income and presence of smoker in the family was sought. When factors affecting affirmative response were explored, education came out to be a significant variable (p = 0.02). When predictors for knowledge regarding specific health effects like cancer, lung and heart disease and women's disease were analyzed, again educated women had better knowledge of lung and women's diseases but not cancer and heart diseases. Education was found to be strongly associated with affirmative response for knowledge about health effects of smoking on fetus (p < 0.001).

**Table 2:** Knowledge towards smoking and its associated factors among women attending primary care clinics in Karachi, Pakistan

Associated Factor	**Q1**.	**Q2**.	**Q3**.	**Q4**.	**Q5**.	**Q6**.	**Q7**.	**Q8**.	**Q9**.
	n (%)	n (%)	n (%)	n (%)	n (%)	n (%)	n (%)	n (%)	n (%)
**Age group**									
- 18 - 25 years	92 (84)	15 (14)	7 (6)	35 (32)	88 (81)	97 (89)	27 (25)	18 (17)	67 (62)
- 26 - 35 years	63 (85)	7 (10)	5 (7)	25 (34)	56 (76)	66 (89)	11 (15)	16 (22)	36 (49)
- >35 years	56 (84)	13 (19)	6 (9)	22 (33)	49 (73)	58 (87)	13 (19)	14 (21)	43 (64)

**Educational status**									
- No formal education	96 (77)*	14 (11)	12 (10)	35 (28)*	83 (67)**	101 (81)*	27 (22)	26 (19)	70 (57)
- Up to 5 years of schooling	31 (91)	7 (21)	3 (9)	16 (47)	32 (94)	31 (91)	8 (23)	2 (6)	24 (71)
- 6 - 10 years of schooling	61 (92)	8 (12)	3 (4)	18 (27)	58 (88)	63 (95)	13 (20)	13 (27)	38 (58)
- >10 years of schooling	23 (89)	6 (23)	0 (0)	13 (56)	20 (77)	26 (100)	3 (12)	7 (27)	14 (54)

**Monthly income (Pak Rupees)**									
- Up to 5000	134 (84)	21 (13)	10 (6)	49 (31)	130 (82)	142 (89)	30 (19)	33 (21)	94 (59)
- >5000	51 (88)	9 (15)	7 (12)	23 (40)	44 (76)	50 (86)	9 (16)	11 (19)	37 (64)

**Smoker in the house**									
- Yes	63 (84)	8 (11)	6 (8)	31 (41)	56 (75)	62 (81)	9 (13)	10 (16)	41 (55)
- No	148 (84)	27 (15)	12 (7)	51 (30)	137 (78)	160 (91)*	41 (23)	36 (21)	105 (60)

• *p value < 0.05, ** p value of <0.005

Q1.Is smoking harmful?

Q2. Does smoking cause cancer?

Q3. Does smoking cause heart diseases?

Q4. Does smoking causes lung disease?

Q5. Does smoking have ill effects on fetus?

Q6. Does passive smoking have ill effects on children?

Q7. Does passive smoking cause lung disease in children?

Q8, Does smoking has bad influence on children?

Q9, Does occasional smoking have ill effects?

We analyzed predictors for knowledge about effects on children and again found education (p < 0.01) to be a significantly important variable. Also having a smoker in the house may predict having better knowledge for lung disease (p = 0.06). Having a higher income or advanced age was not associated with knowledge of adverse effects.

Perceptions about smoking and its associated factors are described in table 3. About half of the women (48%) believed that it helped reduce weight. About one third (38%) of them believed it reduced stress and boredom and 40% women felt it helped one to relax. More women without any formal education had this belief that smoking relieves boredom compared to educated women (p = 0.04) while women having a smoker in the house believed that smoking relieves stress (p = 0.03).

**Table 3:** Perceptions towards smoking and its associated factors among women attending primary care clinics in Karachi, Pakistan

Associated factors	Smoking relieve boredom (P1)	Smoking cause relaxatio n P2	Smokin g relieve stress P3	Smokin g help in losing weight P4
	n (%)	n (%)	n (%)	n (%)
**Age groups**				
- 18 - 25 years	37 (34)	37 (34)	37 (34)	47 (43)
- 26 - 35 years	29 (39)	31 (42)	27 (37)	37 (50)
- >35 years	32 (48)	32 (48)	30 (45)	35 (52)

**Educational status**				
- No formal education	59 (48)*	54 (44)	53 (43)	60 (48)
- Up to 5 years of schooling	11 (32)	11 (32)	8 (24)	16 (47)
- 6 - 10 years of schooling	22 (33)	23 (35)	23 (35)	33 (50)
- >10 years of schooling	6 (23)	12 (46)	10 (39)	10 (39)

**Monthly income (Pak Rupees)**				
- Up to 5000	67 (42)	62 (39)	56 (35)	80 (50)
- >5000	21 (36)	25 (43)	25 (43)	26 (45)

**moker in the house**				
- Yes	36 (48)	32 (43)	36 (48)*	31 (41)
- No	62 (35)	68 (39)	58 (33)	88 (50)

*p value < 0.05

P1. Does smoking relieve boredom?

P2. Does smoking cause relaxation?

P3. Does smoking relieve stress?

P4. Does smoking help in losing weight?

## Discussion

Majority of the studied women recognized smoking to be harmful to women's health but only a small proportion could identify the actual type of effects. The most widely cited adverse effect being lung problems. Other studies have also identified lung problems being the most cited adverse effects of smoking by women [22,24]. This is most likely because of aggressive media coverage on the effects of smoking on the lungs. However the knowledge of smoking causing heart disease was very poor. This is consistent with a study done by Khan JA et al on hospital attendants in Pakistan [24]. This is particularly pertinent to Pakistani women as the prevalence of heart disease is high in Pakistani women [25]. Therefore the relationship between smoking and heart disease in context to women's health needs to be addressed.

This study also revealed that none of the women were aware of the association of smoking with infertility and osteoporosis. This is an important finding as both infertility and osteoporosis are important health problems prevalent in this part of the world and smoking is well known preventable cause for these conditions. This finding is concordant with a US study on hospital employees which found a significantly high proportion of women being unaware of the association between cigarette smoking with infertility and osteoporosis, the latter population being educated (all of them could read) and worked as staff of a hospital [22].

Smoking during pregnancy may cause preterm delivery, low birth weight and sudden infant death syndrome [8]. Majority of studied women identified the link between smoking and poor fetal outcomes but the specific effects were vaguely described and reported. Very small proportion of women could identify the specific effects of passive smoking on fetus. This is consistent with another study on pregnant women who also were unaware of the fetal risks associated with passive smoking [23]. Our study sample was comparatively more aware than the rural Pakistani women studied by Ali S et al where only 6% of the women though that smoking may be harmful to the fetus [21]. This difference of level between urban and rural Pakistan can be explained by comparatively higher educational level of women in cities of Pakistan. Roth et al found a large number of women in the reproductive age groups to be aware of the smoking related pregnancy complication and a very important factor for this increased awareness is the aggressive public health campaigns in United States and discussion during antenatal visits by the medical staff [22]. This reiterates the emphasis on dissemination of information through multiple modalities.

Children's exposure to passive smoking is associated with a range of adverse health outcomes for children [26]. Parental smoking is the most common source of children's exposure to passive smoking. In our study about one third of the participants revealed having at least one (up to maximum of four) smokers in their homes; these smokers are exposing 269 children to passive smoking, most of the smokers being fathers of these children. Bloch et al has also reported 51.4% of children being exposed to passive smoking in a large sample of pregnant women in Pakistan [27]. Knowledge of harmful effects is the key determinant in reducing the passive smoking exposure of children/women as the legislations cannot reach the home, which is the primary place of passive smoking for women and young children [11]. A large number of women in our study recognized passive smoking to be harmful for children's health, though knowledge of specific effects was limited. It has been shown that educating parents about health risks of SHS would significantly reduce exposure to passive smoking [16]. Educating parents about specific effects on their children's health may prompt them to reduce the children's exposure by not smoking indoors or in front of the children. Parents also serve as role models for children and therefore it is likely that a lot of these children who are exposed to passive smoking at home would take up smoking later in their lives. This concern was expressed by the participants of our study and it is well founded by research that there is a strong association between smoking in adolescence and parental smoking. A study from Karachi reported that adolescents are more likely to smoke if any of their parent smoked [28].

Education was associated with better knowledge of adverse effects. This is consistent with other studies showing significant difference in knowledge regarding active and passive smoking [15,18] with respect to education. Interestingly women belonging to households without a smoker were also significantly better educated than women with a smoker in the house (76% vs. 23%).

Our study showed that most of the women thought that smoking is not justified and they disliked being around smokers. Probable reasons of disapproval may be because of increased economic burden of smoking on the household already having limited income. Also parental smoking serves as role modeling for the children besides giving increased access to tobacco products. Smoking also conveys the macho and male building image which may make women uncomfortable. Other reasons of dislike may the unpleasant smell of the smoke. This can be used for building specific interventions based on nullifying the image that is created in the minds of smokers. It is also important to negate the fallacy that was reported by the women in our study on the beneficial effects of smoking like losing weight, relieving stress and to relieve boredom as this is commonly believed that smoking helps lose weight. Similar perceptions have been reported earlier. Women have been shown to use smoking as a means to attain and maintain weight [29]. It is important to understand and work toward addressing these attitudes as it increases the chances of tobacco uptake especially in young girls [30].

Health professionals have a key role in educating women about the dangers of smoking and disproving the myths related to it. Doctor's beliefs and attitudes towards smoking can influence their judgments towards discussing smoking cessation with their patients [31]. Personal use of tobacco may serve as negative role modeling for the patients and may prove as a barrier for discussion A recent study at a hospital in Lahore, Pakistan revealed one third of the doctors to be smokers [32].

There are certain limitations of our study. The study was conducted in three clinics catering to women living in slums hence cannot be generalized to all women of Pakistan. Smoking is a socially and culturally unacceptable habit in this part of world particularly among women, hence underreporting cannot be excluded. One of the limitations of the study is to test many relationships which may inflate the level of significance of different variables as they were not corrected for multiple comparisons. Though prevalence of non-smoke tobacco may be higher, we did not enquire about non-cigarette tobacco smoke as our study was focused on knowledge of health effects of active and environmental tobacco smoking on women and children.

## Conclusions

Nevertheless, the results of this study are sufficient enough to conclude that women were are of the general ill effects of smoking but fail to identify smoking to be associated with female maladies. This may be because the tobacco educational campaigns have mainly focused on risks for men and specific adverse effects on women's health have not been highlighted. Therefore women do not associate themselves with these health problems and considers them a men's health issue.

To better educate the women of health effects of tobacco, anti-tobacco educational strategies and interventions need to be devised. These interventions should be comprehensive, multi-faceted and multi-sectorial involving primary health care providers, public health practitioners, policy makers, social scientist and media. In addition, educational level and status of women needs to be improved and upgraded. There needs to be a plan to disseminate information through mass media or by health warning labels on tobacco products. Health care providers can play a critical role in health education by providing information on effects of active as well as passive smoking on women and children as well as discuss the myths associated with smoking with their patients. Mandatory health warnings on cigarette packages are a form of health information to alert the public to the dangers of smoking and the use of tobacco. But usually tobacco warnings lack the gender specificity therefore adverse effects of passive smoking on women; fetus and children should also be part of the labels. Use of pictures with graphic depictions of disease and other negative images has greater impact than words alone, and is critical in reaching the large number of people worldwide who cannot read as been seen in many countries. The decision of Pakistan government [33] in introducing pictorial warning by 2010 should have positive impact in educating the public.

Public health educational campaigns should include multiple modalities to reach the target audience; like media campaigns on television, radio, bill boards etc and community programs in schools, community and religious institutions. The harmful effects of smoking specific to women, needs to be part of the educational campaigns. Finally physician's role in health education promotion cannot be over emphasized. Women at antenatal clinics should not only be asked about personal use of tobacco but also asked about passive smoking exposure at home and educated about the adverse effects of both. Similarly physicians should ask about passive smoking exposure of children particularly in children with respiratory problems and educate the parents about the effects of passive smoking on children's health.

## Competing interests

The authors declare that they have no competing interests.

## Authors' contributions

SB and MA conceived and designed the study and drafted the manuscript. FT collected, cleaned, entered and validated the data. SB designed the study questionnaire, and analyzed and interpreted the data. AKK critically reviewed the data analysis and interpretation, contributed to the revisions of the manuscript and provided the conceptual feedback throughout. All authors have read and approved the final version of the manuscript.

## Pre-publication history

The pre-publication history for this paper can be accessed here:

http://www.biomedcentral.com/1472-6874/11/16/prepub
